# The Implementation Effectiveness of a Freely Available Pediatric Cancer Pain Assessment App: A Pilot Implementation Study

**DOI:** 10.2196/10280

**Published:** 2018-12-21

**Authors:** Perri R Tutelman, Christine T Chambers, Jennifer N Stinson, Jennifer A Parker, Melanie Barwick, Holly O Witteman, Lindsay Jibb, Hayley C Stinson, Conrad V Fernandez, Paul C Nathan, Fiona Campbell, Karen Irwin

**Affiliations:** 1 Dalhousie University Halifax, NS Canada; 2 IWK Health Centre Halifax, NS Canada; 3 University of Toronto Toronto, ON Canada; 4 The Hospital for Sick Children Toronto, ON Canada; 5 Laval University Quebec, QC Canada; 6 Ottawa Hospital Research Institute Ottawa, ON Canada; 7 University of Ottawa Ottawa, ON Canada; 8 Cancer Knowledge Network Milton, ON Canada

**Keywords:** cancer pain, pain assessment, pediatric cancer, mHealth, eHealth, implementation

## Abstract

**Background:**

Pain Squad is an evidence-based, freely available iOS app designed to assess pain in children with cancer. Once research-based technologies such as Pain Squad are validated, it is important to evaluate their performance in natural settings to optimize their real-world clinical use.

**Objective:**

The objective of this study was to evaluate the implementation effectiveness of Pain Squad in a natural setting.

**Methods:**

Parents of 149 children with cancer (aged 8-18 years) were contacted to invite their child to participate. Participating children downloaded Pain Squad on their own iOS devices from the Apple App Store and reported their pain using the app twice daily for 1 week. Participants then emailed their pain reports from the app to the research team and completed an online survey on their experiences. Key implementation outcomes included acceptability, appropriateness, cost, feasibility, fidelity, penetration, and sustainability.

**Results:**

Of the 149 parents contacted, 16 of their children agreed to participate. More than a third (6/16, 37.5%) of participating children returned their pain reports to the research team. Adherence to the pain assessments was 62.1% (mean 8.7/14 assessments). The 6 children who returned reports rated the app as highly feasible to download and use and rated their overall experience as acceptable. They also reported that they would be willing to sustain their Pain Squad use over several weeks and that they would recommend it to other children with cancer, which suggests that it may have potential for penetration.

**Conclusions:**

While Pain Squad was well received by the small number of children who completed the study, user uptake, engagement, and adherence were significant barriers to the implementation of Pain Squad in a natural setting. Implementation studies such as this highlight important challenges and opportunities for promoting the use and uptake of evidence-based technologies by the intended end-users.

## Introduction

Pain is a prevalent symptom experienced by children with cancer [1-4]. For children with cancer, pain can result from a variety of sources (eg, treatments, procedures, the disease itself) [5-7], and when undermanaged, can have deleterious impacts on many domains of their health and functioning [8-12]. Significant resources have been dedicated to the development and validation of tools to assess and treat pain in children with cancer. These include physical and psychological interventions [13-15], symptom assessment scales [16-18], and mobile health (mHealth) apps [19-22].

**Figure 1:**
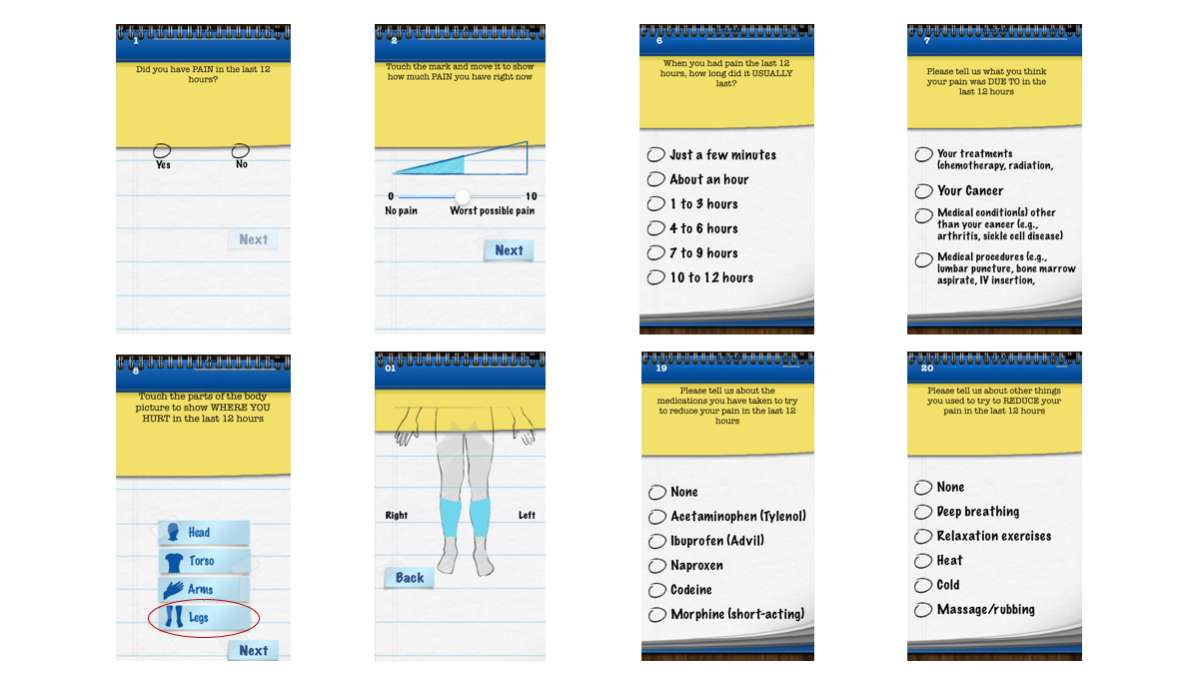
Screenshots of the Pain Squad Pain Assessment (note that users can scroll for additional answer options for the multiple choice questions).

One mHealth tool developed specifically to assess pain in children with cancer is Pain Squad, a gamified pain assessment mobile phone app [19,23]. Pain Squad enables children and adolescents to report their pain twice daily on an iOS device (eg, iPhone or iPad) in real-time by responding to a 22-item multidimensional pain assessment [19]. The pain assessment includes questions on pain intensity, interference, duration, location, and pain management strategies used (Figure 1). Within the app, users play the role of law enforcement officers and are promoted to various ranks based on adherence. Pain reports are stored locally on the user’s device and can be downloaded or emailed to their health care professional. Stinson et al developed the app using a comprehensive user-centered design approach [23] and subsequently evaluated its psychometric properties in 106 children and adolescents with cancer. Pain Squad was found to be a valid, reliable, and feasible pain assessment device for children and adolescents between the ages of 8-18 years undergoing cancer treatment [19]. While there are over 50 apps for pediatric pain in the Apple App Store [24], Pain Squad is currently the only evidence-based and freely available iOS cancer pain assessment app for children and adolescents.

Despite the rigorous development of tools such as Pain Squad to assess and manage pain in pediatric oncology, symptom audits reveal that as many as 92% [25] of children with cancer have pain, and many do not benefit from the best available evidence-based approaches to pain care [26]. This phenomenon, known as the knowledge-to-action gap, refers to failure of the translation of the best available research evidence to be used in regular clinical practice [27]. Knowledge-to-action gaps have been described in many areas of medicine and health, and the availability of mHealth tools shown to be valid and reliable in research studies is no exception. In fact, a 2014 systematic review found that none of the 34 pain apps published in peer-reviewed journals were available to end-users [28] (since the time of the review, Pain Squad has become the only freely available pediatric pain iOS app). Apps like Pain Squad, which aim to measure patient-reported outcomes and experiences to better tailor care to each patient, are of little clinical use if they are not used by patients. Failure to ensure uptake is a barrier to the provision of evidence-based care [29].

Implementation science has emerged as a field of study to better understand uptake of new interventions. Defined as, “the scientific study of methods to promote the systematic uptake of research findings and other evidence-based practices into routine practice” [30], implementation science seeks to identify theories, processes, strategies, and outcomes to enable the use of evidence-based practices in natural contexts. While efficacy studies of pain management tools traditionally measure clinical outcomes to assess the performance of the intervention (eg, participant pain, quality of life, functional disability) [19,20,31], implementation studies evaluate outcomes associated with the performance of the tool in a real-world setting (eg, acceptability, adoption, cost, penetration) [32,33]. Pain Squad’s validity as a pain assessment tool in pediatric oncology has been previously evaluated in tightly controlled research studies [19,23]. However, research examining the implementation effectiveness of Pain Squad is needed to determine its performance in natural settings and to guide and promote its uptake into routine pediatric oncology practice. Thus, the objective of this study was to evaluate the implementation effectiveness (ie, acceptability, appropriateness, cost, feasibility, fidelity, penetration, and sustainability) of the Pain Squad app in a naturalistic context.

## Methods

### Participants

We accessed child participants by contacting parents who had participated in a larger online project [7] and consented to be contacted for future research (n=149). These parents received an invitation for the current study via email. Their children were eligible to participate if they (1) were between the ages of 8-18 years, (2) had a history of cancer, (3) were currently undergoing cancer-related treatment or were a cancer survivor, (4) had experienced any pain in the past week, (5) had a personal iOS device, and (6) could read and understand English. There were no geographic restrictions. One reminder email was sent 48 hours after the initial invitation.

### Procedure

**Figure 2:**
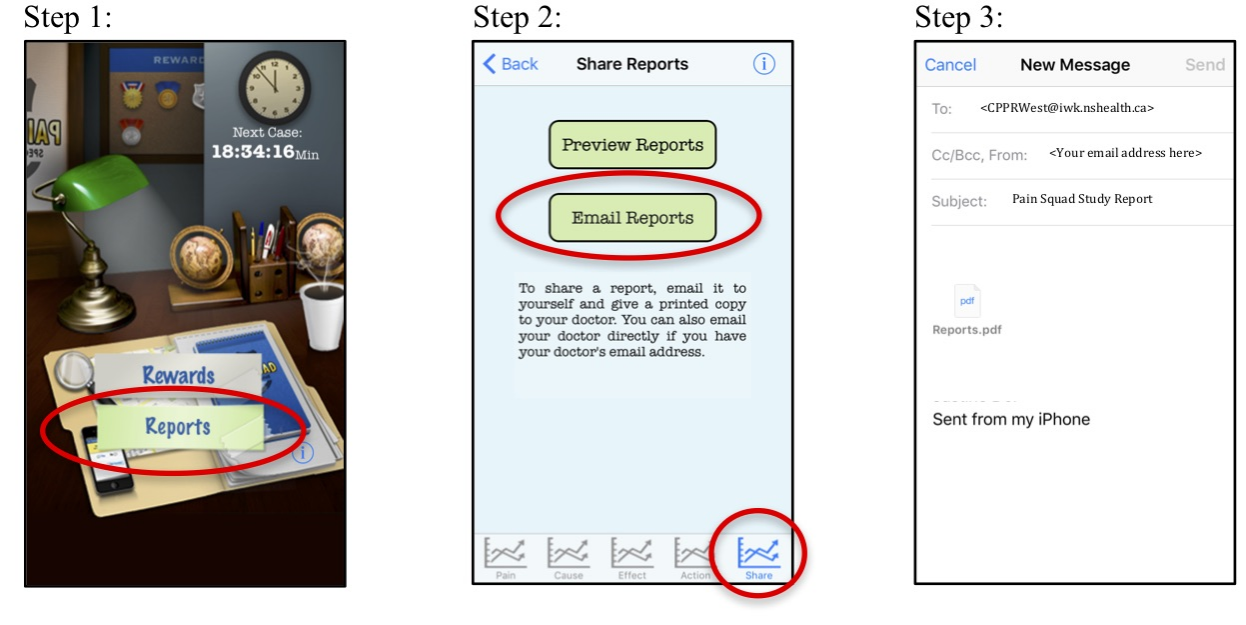
Screenshots from the publicly available version of Pain Squad demonstrating the process of emailing a pain report.

Parents who replied to the invitation email expressing interest in participating were emailed a consent form and an investigator-developed document with information for their child on how to use Pain Squad. Children were asked to download the app from the iTunes store onto their iOS device and complete pain assessments twice daily on the app for a minimum of 1 week. Users are able to customize the timing of their pain assessments within the app to coincide with their schedules, so long as the assessments are scheduled 12 hours apart. Pain Squad sends users push notifications to their device at the time of their scheduled reports, after which they have 30 minutes to complete the report or else it is counted as missed. The app was designed this way by the developers to capture children’s pain assessments in real-time and reduce the impact of recall bias [19,23]. In the Pain Squad information document, participants were reminded to ensure their notification settings were turned on for Pain Squad to ensure they received the reminders to complete the reports. This study used the publicly available version of Pain Squad that stores all data directly on the individual’s device. Thus, participants were required to use the built-in email feature to send their pain report to the research team after the testing period (Figure 2). Previously published studies using Pain Squad used the research version of the app, which has server connectivity providing researchers with direct access to participants’ pain assessments, and provided participants with study iOS devices preloaded with the Pain Squad app [19] (the team that developed the app removed server connectivity from the public version of the app for data security reasons). Children in this study were required to download the app onto their own devices mimicking the realistic end-user experience. Participants were reminded via email midway through the week to continue using the app and to submit their reports at the end of the week. Participants who did not submit their reports were sent two follow-up emails. Two months later, children and their parents who submitted reports were emailed a link to a follow-up survey to collect their demographic information and ask about their experience using Pain Squad. Children who submitted pain reports received a Can $25 gift card to an online retailer. Those who completed the follow-up survey were entered into a prize draw to win an additional Can $25 gift card. This study was approved by the institutional research ethics board of the IWK Health Centre, Nova Scotia, Canada.

### Measures

#### Participant Recruitment, Retention, and Adherence

Recruitment was evaluated by the response rate to the invitation email and proportion of participants agreeing to participate. Retention was assessed based on the proportion of final reports received by the research team, and adherence was assessed as the proportion of pain assessments completed of a possible 14 (two reports daily for 7 days).

#### Implementation Effectiveness

Participants answered 10 questions (rated on a scale of 1-5) to assess key implementation outcomes including acceptability (ie, participant satisfaction), appropriateness (ie, perceived usefulness), feasibility (ie, utility), penetration (ie, spread), and sustainability (ie, maintenance) as described in Proctor’s taxonomy [32]. Questions were adapted from the Acceptability E-Scale (AES), a 6-item questionnaire that assesses the acceptability of electronic self-report symptom tools on a 5-point scale [34]. The AES has been found to be valid and reliable and has been used previously with pediatric oncology populations [31,35]. Two questions were derived from the acceptability questionnaire used by Jibb et al in a pilot study of the Pain Squad+ app [31], and two questions were developed by the research team. Consistent with published cut-off values for the AES, a mean score >3 on any item indicated a positive evaluation [34] and scores ≥4 were considered high [35]. Other implementation outcomes including cost and tool fidelity (ie, technical difficulties reported by participants, use of the app as intended) were assessed by the research team.

#### Open-Ended Questions

Two open-ended questions (“What was your favorite part about using the Pain Squad app?” and “What was your least favorite or the most challenging part about using the Pain Squad app?”) were included in the follow-up survey.

#### Demographic Information

In the follow-up survey, parents were asked to report on their child’s date of birth, sex, cancer diagnosis, time since diagnosis, country of residence, remission status, and ethnicity.

### Data Analysis

Descriptive statistics including frequencies, means, standard deviations, and ranges summarized the quantitative data. Content analysis was used to summarize the qualitative responses according to the procedure outlined by O’Cathain and Thomas [36].

## Results

### Participant Recruitment, Retention, and Adherence

**Figure 3:**
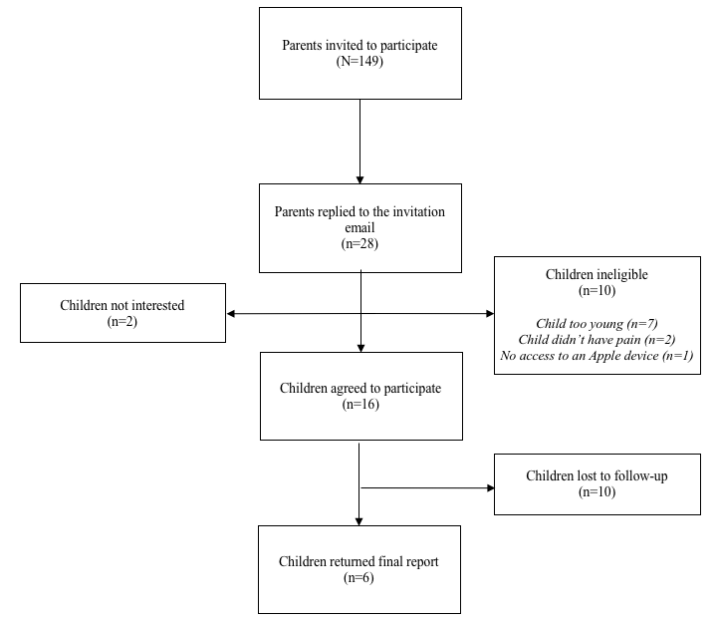
Flow of participants through the study.

Of the 149 parents invited to participate, 28 parents (18.8%) replied to the email, of whom 16 (57.1%) of their children agreed to participate. Ten of the 28 parents who replied to the email indicated that their children were ineligible to participate due to their age (n=7), lack of pain in the past week (n=2), and no access to an iOS device (n=1). Two parents replied to the email indicating that their children were not interested. After the 1-week period, more than a third of participants (6/16, 37.5%) returned their reports to the research team. Figure 3 depicts the flow of participants through the study. A total of 52 pain assessments were completed by the 6 participants. Out of a possible 14 assessments per child, children completed an average of 8.7 (62.1%) assessments (SD 4.18, range 4-15). One participant completed more than the minimum amount of reports (a total of 15). As described in Table 1, the 6 children who returned their Pain Squad reports were almost all female and in remission (information on any current disease directed therapies was not available). Children ranged in age from 8-17 years old and resided in Canada (n=2), the United States (n=2), and the Netherlands (n=2). One participant was diagnosed 1-2 years prior to participating, 4 participants were diagnosed 2-5 years prior to participating, and 1 participant was diagnosed 5-10 years prior to participating. All parents identified their children as Caucasian.

**Table 1:** Characteristics of participants who returned reports.

Participant	Age, years	Sex	Diagnosis	Remission	Completed reports, n^a^
1	15	Female	Osteosarcoma	Yes	8
2	8	Female	ALL^b^	No	8
3	17	Female	Germ cell tumor	Yes	5
4	14	Male	ALL^b^	Yes	15
5	9	Female	ALL^b^	Yes	4
6	13	Female	Brain tumor	Yes	12

^a^14 possible reports.

^b^Acute lymphoblastic leukemia.

### Implementation Effectiveness

A summary of the participants’ ratings on key implementation outcomes are provided in Table 2. Overall, participants provided positive evaluations for 9 of the 10 outcomes and high evaluations for 5 of those outcomes. Participants rated Pain Squad as being highly feasible to download and use and rated their experience using it as acceptable. Ratings of the app’s appropriateness varied. Participants rated the app’s helpfulness with describing pain positively, although the average rating of the app’s helpfulness with treating pain was evaluated negatively. Participants’ responses demonstrated a potential for wide penetration of the app, reporting on average that they would be highly likely to recommend Pain Squad to another child with cancer. In terms of sustainability, one participant reported that they would be willing to use it for the same amount of time, while the others indicated that they would be willing to use it for longer. There was no direct cost associated with implementing Pain Squad as it is freely available on the Apple App Store. With respect to fidelity, no participants reported any major technical issues using the app. However, difficulty did occur when participants tried to download the app on an iPad (as opposed to iPhone), as an extra step was required in the Apple App Store interface. The app was also not used entirely as intended by participants. As mentioned above, only 6 of 16 participants returned reports to the research team, and on average, fewer than the 2 required pain assessments were completed per day. Also, it was noted that 2 participants reported higher levels of pain for their “least” compared to their “worst” pain scores in a pain assessment (pain data not reported).

### Open-Ended Responses

Five participants (5/6, 83%) provided comments in the open-ended questions of the follow-up survey.

#### Favorite Part of Using Pain Squad

Regarding participants’ favorite part of using Pain Squad, 3 participants discussed the app’s features and usability.

The rewards and the “promotions.” Thought it was cool to be “promoted.”

It was very easy to fill in and did not take a lot of time.

One participant acknowledged the app’s usefulness as their favorite feature:

I liked all of the questions the app asked me, they helped describe my pain, which is pretty hard.

#### Least Favorite or Most Challenging Part of Using Pain Squad

Timing of the assessments necessitated participants having their iOS device with them, and this was identified as the least favored or most challenging part of the Pain Squad experience by 2 participants. For instance, participants described:

Being in the right place at the right time was sometimes difficult – remembering as well. My phone is not always with me.

**Table 2:** Survey questions evaluating key implementation outcomes.

Question			Associated implementation outcomes		Answer choices		Possible scores, range		Actual scores, range		Mean score (SD)		n
**Items from the Acceptability E-Scale**													
	How easy was Pain Squad for you to use?	Feasibility		N/A^a^		1-5		3-5		4.17 (0.75)		N/A	
	How understandable were the questions?	Acceptability		N/A		1-5		3-5		3.83 (0.75)		N/A	
	How much did you enjoy using Pain Squad?	Acceptability		N/A		1-5		2-5		3.67 (1.20)		N/A	
	How helpful was Pain Squad in describing your pain?	Appropriateness		N/A		1-5		1-5		3.50 (1.38)		N/A	
	Was the amount of time it took to complete Pain Squad acceptable?	Acceptability		N/A		1-5		4-5		4.67 (0.52)		N/A	
	How would you rate your overall satisfaction with Pain Squad?	Acceptability		N/A		1-5		4-5		4.33 (0.52)		N/A	
**Items from Jibb et al**													
	How helpful was Pain Squad in treating you pain?	Appropriateness		N/A		1-5		1-4		2.33 (1.03)		N/A	
	How long would you be willing to use Pain Squad?	Sustainability		Same amount of time		N/A		N/A		N/A		1	
				2 weeks		N/A		N/A		N/A		1	
				4 weeks		N/A		N/A		N/A		0	
				6 weeks		N/A		N/A		N/A		1	
				≥8 weeks		N/A		N/A		N/A		3	
**Investigator-developed items**													
	How easy was it to download Pain Squad from the App store to your device?	Feasibility		N/A		1-5		3-5		4.00 (0.90)		N/A	
	How likely would you be to recommend Pain Squad to another child with cancer?	Penetration		N/A		1-5		4-5		4.33 (0.52)		N/A	

^a^N/A: not applicable.

The tricky thing was the timing. It was done during the summer and sometimes we were out or doing things at the time I had to fill it in. It was hard to always be at the iPad when it was time to fill it in. I do not have a phone, so I had to be at my house to do it. It was hard.

Two participants commented on the commitment required. The following quote illustrates this point:

My daughter disliked that she had to fill in the questions daily.

One participant expressed dislike for a feature in the app:

I didn’t like that some options didn’t have a back button.

Finally, one participant described that the app would have been more relevant and useful at a different stage of their disease:

It would have been great to have had this while in treatment to record pain as there was a lot then.

## Discussion

### Principal Findings

The objective of this study was to evaluate the implementation effectiveness of Pain Squad, a free evidence-based cancer pain assessment app, in a naturalistic context. Results provide preliminary data to support the use of Pain Squad as a pain assessment tool for children with cancer who have access to an iOS device. However, the study also identified significant barriers and challenges associated with the app’s use and uptake by end users.

Similar to what has been reported in other mHealth studies [37,38], we encountered challenges with participant recruitment and retention in this naturalistic context outside of a traditional clinical research trial. Of 149 parents who were invited to participate, 16 agreed for their child to participate, and only 6 had children who completed the study by sending in their pain assessment report at the end of the testing period. This is in contrast to the Pain Squad validation trial, which recruited 92 children and adolescents with cancer and managed to retain all enrolled participants throughout the course of the study [19]. We surmise some key contextual differences may account for these differences. First, the current study recruited and engaged with participants entirely online. Indeed, it is unclear how many of the 149 parents contacted had children meeting the study’s eligibility criteria. While emerging research suggests that online recruitment may be advantageous [39], lack of in-person contact is a well-documented limitation of mHealth studies and may negatively influence recruitment, retention, and effectiveness [40]. This challenge was likely compounded in this study by the need to access child participants via their parents. Second, unlike the research version of the app used in past usability, feasibility, and validation studies [19,23], the public version of Pain Squad used in this study does not feature any network or server connectivity, requiring participants to email their final pain assessment report to the research team. Participants did not raise this as a challenge in the open-ended questions; however, it is possible that this extra step was a barrier for study completion and restricted our ability to collect partial data from participants who started but did not complete the study. Future public versions of Pain Squad should consider the possibility of server connectivity to allow clinicians to access users’ pain data without requiring this additional step, or alternatively, adding other ways for users to send in their reports, such as text message, which is a communication method more commonly used by children and adolescents [41]. These contextual differences reflect real-world issues that children downloading and using Pain Squad may encounter in their everyday lives outside of tightly controlled and well-resourced research trial environments. These are challenges that should be considered by the app development community, which may wish to evaluate the differential effectiveness of various app features (ie, report submission via text message). This could be done in a sample of healthy children to prevent undue burden on vulnerable medical populations.

Reporting adherence varied significantly for the 6 participants who submitted report data, ranging from 4-15 assessments. Overall, the average adherence rate in this study was lower than the rate of adherence in previous Pain Squad feasibility and validation studies [19,23]. This “voltage drop,” whereby the success of a tool decreases once it is tested in naturalistic settings, has been previously described [42]. This decrease in adherence may be related to the fact that completing pain assessments within 30 minutes of the scheduled time may be difficult to attain or sustain for many children and families but may also reflect other important differences, such as the characteristics of the community-based sample. Hardiker and Grant [43] reviewed factors that influence public engagement with electronic health (eHealth) and found that among adults, engagement with eHealth can vary significantly based on individual characteristics including age, disease severity, motivation to improve one’s own health, and the belief that the intervention will improve one’s health [43]. The majority of children in our study were in remission, and while pain can remain an issue for childhood cancer survivors, it is often significantly lower in intensity than children who are in active treatment [7,44]. Thus, addressing pain with the app presented lower relative advantage for the participants—a construct described in the Consolidated Framework for Implementation Research [45]. Children in Stinson et al’s validation study [19] were in active treatment, and thus, the opportunity for buy-in, relative advantage, and tension for change [45] were presumably more compelling. It is important to note that in both the original validation study [19] and the current implementation pilot, participants’ submitted pain reports were used for research purposes only. This lack of potential for improved clinical care may also afford lower relative advantage and is a point of ecological validity that should be addressed in future implementation studies of Pain Squad. Finally, participants in this study were offered a monetary incentive to complete the pain assessments and return their final report to the research team, and this may have been a key feature driving compliance [46,47]. When the app is used in a clinical setting without monetary incentives, researchers and clinicians may experience an additional drop in retention and adherence. Future studies using Pain Squad may wish to conduct a process evaluation to better understand barriers to participant retention and adherence. This may reveal additional challenges to be considered (eg, preferences for alternative gamification themes or need for further information on the benefits of using the app).

Participants’ comments about their experiences using Pain Squad were generally positive (Table 2). They reported the app as acceptable, feasible, sustainable, and having potential for broad penetration. Most participants (5/6) reported they would be willing to use Pain Squad for an extended period of time; however, they described the limited time window for completing pain assessments and the commitment required as challenges. These challenges were also identified by participants in the Pain Squad validation study [19]. Future versions of Pain Squad should take this feedback into account to promote adherence. Further, while the current study was only 1 week in duration, the gamification element of the app terminates after a 2-week period (ie, participants can achieve the highest law enforcement rank after completing 2 weeks of pain assessments). Future versions of the app should also consider additional incentives for longer-term use. Participants’ perceptions of the appropriateness of the app were mixed. Participants reported that the app was helpful for describing their pain, but not for treating their pain. This version of Pain Squad collects data on the strategies that participants select to treat their pain but was designed primarily as a pain assessment tool. An enhanced version of Pain Squad, Pain Squad+, provides real-time pain management recommendations according to a standardized pain treatment algorithm in response to pain reported in app assessments and was able reduce pain intensity and interference in an efficacy pilot with 40 children [31]. Building on the results of the current study, it will be important to conduct implementation studies of Pain Squad+ after its effectiveness is demonstrated to optimize its relevance to children with cancer in real-world settings.

### Limitations

This work is not without limitations. First, the results of this study describe the experiences of a small number of children with cancer (most of whom were in remission) whose perspectives and experiences may not be representative. As well, the study eligibility criteria required children to have access to an iOS device, which introduced sample selection biases based on socioeconomic status and participants’ device brand preferences. Further, the study was limited to the perspectives of the main app user, children with cancer. Future implementation studies should evaluate the perspectives of other users involved such as parents, regarding their role in implementing the intervention, as well as clinicians and organizational administrators to assess other potential barriers and facilitators to the implementation of Pain Squad in clinical settings [48]. The findings of this pilot work could be used to adapt Pain Squad for a full implementation trial to evaluate the effectiveness of various implementation strategies (eg, advertisements and prescription by health care providers) to achieve optimal dissemination and uptake of the app.

### Conclusions

In conclusion, this study demonstrated that Pain Squad was generally well received by a small sample of children with cancer in a naturalistic context. However, specific challenges related to user engagement and adherence were revealed that are unique to a naturalistic setting. This work highlights the importance of studying implementation outcomes for evidence-based technologies, such as Pain Squad, to optimize their use when made available to the intended end-users.

## Abbreviations

AES: Acceptability Evaluation Scale
eHealth: electronic health
mHealth: mobile health
